# SenseLite: A YOLO-Based Lightweight Model for Small Object Detection in Aerial Imagery

**DOI:** 10.3390/s23198118

**Published:** 2023-09-27

**Authors:** Tianxin Han, Qing Dong, Lina Sun

**Affiliations:** Department of Process Equipment and Control Engineering, School of Mechanical Engineering and Automation, Northeastern University, Shenyang 110819, China; 2270327@stu.neu.edu.cn (T.H.); 2270324@stu.neu.edu.cn (Q.D.)

**Keywords:** small objects, aerial images, YOLOv5, involution, GSConv, SE, Soft-NMS

## Abstract

In the field of aerial remote sensing, detecting small objects in aerial images is challenging. Their subtle presence against broad backgrounds, combined with environmental complexities and low image resolution, complicates identification. While their detection is crucial for urban planning, traffic monitoring, and military reconnaissance, many deep learning approaches demand significant computational resources, hindering real-time applications. To elevate the accuracy of small object detection in aerial imagery and cater to real-time requirements, we introduce SenseLite, a lightweight and efficient model tailored for aerial image object detection. First, we innovatively structured the YOLOv5 model for a more streamlined structure. In the backbone, we replaced the original structure with cutting-edge lightweight neural operator Involution, enhancing contextual semantics and weight distribution. For the neck, we incorporated GSConv and slim-Neck, striking a balance between reduced computational complexity and performance, which is ideal for rapid predictions. Additionally, to enhance detection accuracy, we integrated a squeeze-and-excitation (SE) mechanism to amplify channel communication and improve detection accuracy. Finally, the Soft-NMS strategy was employed to manage overlapping targets, ensuring precise concurrent detections. Performance-wise, SenseLite reduces parameters by 30.5%, from 7.05 M to 4.9 M, as well as computational demands, with GFLOPs decreasing from 15.9 to 11.2. It surpasses the original YOLOv5, showing a 5.5% mAP0.5 improvement, 0.9% higher precision, and 1.4% better recall on the DOTA dataset. Compared to other leading methods, SenseLite stands out in terms of performance.

## 1. Introduction

With the rapid advancement of modern remote sensing technology, aerial imagery has found increasingly widespread applications in sectors such as agriculture, urban planning, and defense. Aerial images offer a unique top-down perspective, allowing for the observation and analysis of vast geographical areas. However, the inherent low resolution and intricate backgrounds of these images pose a technical challenge in accurately detecting small objects [1].

In the realm of conventional machine learning techniques, both support vector machines [2] and decision trees [3] have often been harnessed to pinpoint small entities present in aerial imagery. While these algorithms are straightforward to implement and boast rapid processing times, they hinge on hand-crafted features and exhibit sensitivity to noise. In intricate scenarios, their accuracy often leaves much to be desired, and they fall short in capturing hierarchical features.

In contrast to conventional object recognition techniques, employing deep learning object detection resembles a black-box operation. By simulating the visual sensory mechanism of the human cortex, deep learning architectures directly derive attributes from unprocessed photos, transmitting them sequentially through layers to ascertain the image’s intricate data. This proficiency has instigated a paradigm shift in the domain. Across manifold tasks like categorizing images, partitioning, and pinpointing objects, these frameworks have manifested excellence that greatly exceeds older traditional models [4].

However, the application of standard deep learning models to aerial imagery presents its own set of challenges. The vastness of the scenes, the variability in object scales, and the diverse range of objects represent unique obstacles. Moreover, the dynamic nature of aerial scenes, which is influenced by factors such as changing weather conditions, shadows, and seasonal variations, further complicates the detection task.

A significant challenge in aerial imagery is the detection of small objects, which can be categorized based on their relative size: ultra-small, medium-small, and slightly larger. Ultra-small objects, with limited pixels, are easily overshadowed by background noise. Medium-small objects, sometimes partially obscured, demand complex detection algorithms. Slightly larger objects, which are prevalent in real-world applications, require understanding of their contextual relationship with the environment and might necessitate multiscale detection strategies. Across all sizes, challenges like varied lighting, complex backgrounds, and object similarity further complicate detection. Among the challenges posed by aerial imagery, the detection of small objects stands out as particularly demanding. Due to their reduced footprint in the image, these objects often get overshadowed by vast backgrounds, environmental complexities, and the intrinsic resolution limitations of aerial captures. In the context of this paper, we define an object as ‘small’ if it covers less than 1% of the total image area. Such small objects, despite their size, often hold immense importance in applications like urban planning, traffic monitoring, and military reconnaissance. The precise detection of these entities amidst the vastness of aerial images is the primary focus of our proposed SenseLite model.

Recent advancements have seen the development of specialized deep learning architectures tailored for aerial imagery. These models incorporate modifications to handle the unique challenges posed by aerial data, such as multi-scale object detection mechanisms, robustness to varying lighting conditions, and the ability to handle large-scale datasets efficiently [5]. The integration of attention mechanisms and region proposal networks has further enhanced the precision and recall rates of these models.

However, a significant gap remains in terms of achieving real-time object detection in aerial imagery without compromising accuracy. The computational demands of state-of-the-art models often render them unsuitable for deployment in scenarios requiring immediate feedback, such as drone-based surveillance or real-time traffic monitoring from aerial platforms [4].

Within this paper, we aim to address this problem by introducing SenseLite, a lightweight and efficient model tailored for aerial imagery object detection. Our contributions are:

1. Introduction of Involution, a neural network operation for aggregation of contextual semantic information and adaptive weight distribution;
2. Employment of GSConv, a lightweight convolution method, reducing model complexity while maintaining performance;
3. Integration of a squeeze-and-excitation (SE) attention module for effective exploitation of channel interdependency;
4. Adoption of Soft-NMS to handle overlapping bounding boxes, allowing for retention of multiple valid detections;
5. Introduction of SenseLite, a streamlined model, reducing network parameters by about 30.5%. It surpasses the original YOLOv5 by increasing the mean average precision (mAP) by 5.5%, boosting precision by 0.9%, and increasing recall by 1.4%.

The structure of this manuscript is outlined below. Section 3 examines the research related to aerial remote sensing imagery. Section 4 provides a detailed explanation of our SenseLite approach. In Section 5, we showcase and deliberate upon our experimental outcomes, underscoring the effectiveness of our method. Lastly, Section 6 wraps up this paper and proposes avenues for upcoming research.

## 2. Related Work

In this section, we delve into traditional strategies utilized to identify minuscule entities within aerial photographs, deep learning methodologies, optimized deep learning architectures, and remote sensing applications in aerial imagery.

### 2.1. Traditional Methods for Detecting Small Objects within Aerial Images

Traditional methods for detecting small objects in aerial images include feature-based methods, background modeling methods, and the application of machine learning algorithms. Recently, Hao Zhang and his team introduced a novel adaptive slicing method named ASAHI, which can dramatically reduce redundant computation using an adaptive slicing size. This method has been shown to increase the mAP50 by 0.9% and reduce the computational time by 20–25% on the VisDrone and xView datasets [6].

In another study, Lili Zhang and colleagues proposed a lightweight object detector named Shuffle-GhostNet-based detector (SG-Det) for real-time maritime object detection in UAV images. This method achieved an accuracy rate of over 90% for maritime objects [7].

Furthermore, Xin Wu and associates introduced UIU-Net, a “U-Net in U-Net” framework for infrared small object detection. This method has been shown to be effective in various infrared image scenarios [8].

However, these traditional methods usually have problems such as sensitivity to noise interference, low accuracy, and limited application scenarios. However, these traditional methods usually have problems such as sensitivity to noise interference, low accuracy, and limited application scenarios.

### 2.2. Deep Learning for Small Object Detection within Aerial Images

Deep learning methodologies have been widely utilized to detect small objects in aerial visuals, surpassing conventional techniques in intricate situations. For instance, Cheng Chuanxiang, Yang Jia, and their team introduced an adaptive detection method for aerial survey ground control points based on YOLOv5-OBB. Their method significantly improved small object detection capabilities in images derived from UAVs [9].

Wei Dai and Daniel Berleant explored the limitations of image quality assessments with noised deep learning image sets. Their research emphasized the importance of image quality, especially when detecting small objects in aerial visuals, and how it can affect the overall performance of deep learning models [10].

In another work, Alessandro Betti presented a lightweight and accurate YOLO-like network for small target detection in aerial imagery. This model’s performance in terms of mean average precision (mAP) showcased its potential in real-world applications, where detecting small objects in aerial visuals is crucial [11].

In conclusion, while deep learning approaches have made significant progress in detecting small objects in aerial images, these architectures often require substantial computational resources, making them challenging to deploy in resource-limited environments.

### 2.3. Lightweight Deep Learning Models for Small Object Detection within Aerial Images

The appeal of svelte deep learning architectures in identifying small entities within aerial visuals is anchored in their computational nimbleness. Within the realm of aerial imagery interpretation, these refined frameworks are in increasing demand owing to the operational efficacy they provide.

Hu’s team introduced EL-YOLO, a paradigm fine-tuned for constrained GPU prowess, rectifying the limitations inherent to conventional detection systems. This paradigm distinguishes itself for a triad of salient aspects. It re-examines and crafts three model structures to enhance small object recognition in aerial visuals. The model leverages efficient spatial pyramid pooling (ESPP) to emphasize small object feature subtleties. Moreover, it integrates an α-CIoU loss method to mitigate the bias between favorable and unfavorable samples in skyborne images [12].

Zhao, Lei, and their team proposed an innovative and lightweight method for spotting objects in aerial images, LAI-YOLOv5s, which demonstrated both high accuracy and reduced computational demands [13].

The primary advantage of these lightweight models is their high computational efficiency and small model size, albeit potentially at the cost of some detection accuracy.

Striving for an optimal equilibrium between precision and performance, we unveil the SenseLite framework in this manuscript. This construct endeavors to meld the precision intrinsic to deep learning methods with the computational nimbleness of streamlined models, thereby representing a potent approach for the detection of small entities within aerial visuals.

### 2.4. Remote Sensing Applications in Aerial Imagery

Remote sensing in aerial imagery has evolved significantly, enabling diverse applications. Gang Xu et al. delved into the fusion of compressed sensing and machine learning, enhancing aerial image quality [14]. Maritime target detection, which is a challenge in aerial imagery, has been addressed using weighted sparse approaches, emphasizing precision on complex backgrounds [15].

Innovations also include autofocus-integrated imaging techniques for clarity [16] and feature-enhanced imaging through non-convex–non-local total variation regularization [17]. Additionally, advancements in image reconstruction using compressive sensing have refined aerial imagery quality [18].

These advancements set the stage for our SenseLite framework, which was designed to detect small objects in aerial imagery, leveraging both traditional and modern techniques.

### 2.5. Overview of the Proposed SenseLite Approach

In aerial image analysis, the detection of diminutive objects stands as a non-trivial task due to the intricacies posed by vast backgrounds, environmental factors, and the minuscule nature of the targets. Therefore, we introduce SenseLite, a novel framework tailored explicitly for efficient and accurate object detection in aerial images. SenseLite seeks to amalgamate the essence of deep learning’s detection prowess with the agility of lightweight architectures, fostering high accuracy, even in real-time application scenarios.

At its core, SenseLite employs innovative modifications to the acclaimed YOLOv5 model, integrating cutting-edge techniques like the Involution operator for enriched semantics and improved weight distribution. Balancing computational efficiency and detection performance, components like GSConv and slim-Neck find their integration within the model. Furthermore, the infusion of a squeeze-and-excitation (SE) mechanism catalyzes enhanced channel communication, while the Soft-NMS strategy ensures precision in detecting overlapping targets, even in densely packed aerial visuals.

SenseLite’s inception was driven by the need for a model that can maintain a judicious balance between computational efficiency and detection accuracy. In the next section, we delve deeper into the intricacies of the SenseLite model, uncovering the rationale behind our design choices and showcasing its prowess through rigorous evaluations.

## 3. Modification of YOLOv5

### 3.1. The YOLOv5 Framework

**Figure 1:**
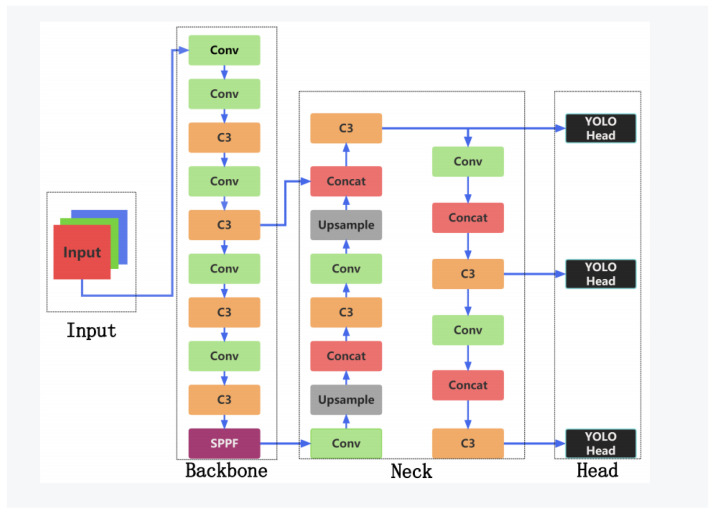
Architecture of the YOLOv5 model.

YOLOv5, an innovation introduced by Bochkovskiy and the Ultralytics team in 2020, represents a significant evolution of its predecessor, YOLOv4 [19]. This iteration, in comparison to its antecedents, boasts a design hallmarked by efficiency and succinctness, especially in terms of feature extraction and handling of scale variance. Melding state-of-the-art architectural design with an optimized loss function, YOLOv5 showcases exemplary performance across diverse computer vision domains. A visual representation of the sophisticated YOLOv5s structure is presented in Figure 1.

**Input:** YOLOv5 places a pronounced emphasis on image quality optimization at the input level. Beyond conventional dimension normalization and pixel value standardization, it incorporates a color metric modulation technique. By transitioning images through varied color spaces, this modulation amplifies the model’s detection capabilities under diverse lighting conditions.

**Backbone:** The backbone of YOLOv5 is underpinned by the design philosophies of deep residual networks (ResNets). This structure synergistically integrates pivotal modules like bottleneck CSP and SPPF for adept feature extraction. Specifically, bottleneck CSP, with its cross-layer connections, enhances the model’s representational power. Simultaneously, the SPPF module, through its spatial pyramid pooling strategy, adeptly captures multiscale features.

**Neck:** Serving as the intermediary between the backbone and the detection head, YOLOv5’s neck integrates a path aggregation network (PAN). This feature pyramid structure is pivotal for efficiently processing objects of divergent scales, especially minuscule targets prevalent in aerial imagery.

**Head:** The head of YOLOv5 accentuates multiscale object detection. It employs multiple scale-specific output layers, each tailored to detect objects within a designated scale spectrum. This meticulous design ensures the model’s robustness and precision across an expansive scale gradient.

While the YOLO algorithm performs well in many computer vision tasks, it struggles with object detection in aerial images, where small objects are set against complex backgrounds. These images demand a model that is both accurate and lightweight for real-time applications like drone surveillance. Unfortunately, YOLO’s computational demands limit its real-time efficiency. To overcome this, we present SenseLite, a streamlined model optimized for aerial image detection.

### 3.2. SenseLite

**Figure 2:**
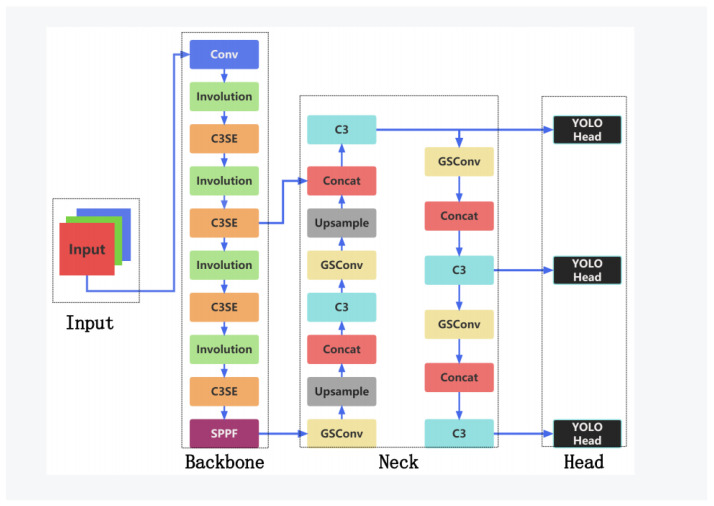
SenseLite model structure.

In this paper, we introduce SenseLite, a pioneering system designed specifically to identify tiny objects in aerial imagery, as depicted in Figure 2. At its core, SenseLite incorporates the *Involution* operation, aggregating contextual semantic information across a broad scope and facilitating adaptive weight distribution. This integration generates multiscale feature maps imbued with rich contextual nuances.

The architecture utilizes GSConv’s efficient convolution methods alongside the Slim-neck approach. This approach maintains performance while significantly trimming the model’s complexity, catering to rapid predictions. Essential to this structure is the *Involution* module, which broadens context, and the squeeze-and-excitation (SE) attention module, optimizing channel interplay.

The SE module, in particular, magnifies the significance of various channels, concentrating the model’s attention on pivotal features and thereby heightening detection precision.

To adeptly handle overlapping bounding boxes, the Soft-NMS technique is integrated, ensuring retention of multiple valid detections—a critical capability, given the potential density of objects in aerial imagery.

In essence, SenseLite is sculpted to harmonize performance and efficiency. By judiciously reducing network parameters and embedding advanced modules like *Involution* and *SE*, it manifests superior detection performance with computational frugality. Subsequent sections elucidate the intricacies of each module and validate the efficacy of SenseLite in practical applications.

## 4. Improvement of the YOLOv5s Algorithm

### 4.1. Soft-NMS

Object detection, a pivotal domain within computer vision, has persistently faced the intricate challenge of accurately identifying and localizing multiple object instances in images. This becomes particularly arduous when objects either overlap or are situated in close proximity to one another. The conventional non-maximum suppression (NMS) technique provides a rudimentary solution to this quandary [20]. However, its efficacy diminishes in scenarios with high object density. In such contexts, traditional NMS may unintentionally discard prediction boxes that, despite having a substantial intersection over union (IoU) with the box of highest confidence, remain crucial for accurate detection. Recognizing this shortcoming, the Soft-NMS method was proposed as a refined and adaptive alternative to traditional NMS [21].

Traditional NMS operates by prioritizing prediction boxes according to their confidence scores. The box with the pinnacle of confidence is chosen as a reference. Subsequent boxes are then evaluated based on their IoU with this reference, and those exceeding a predefined IoU threshold are discarded. The underlying rationale is that a genuine object should ideally be represented by a singular, optimal prediction box. This process is encapsulated by the following equation:

(1) $$\begin{array}{c} s_{i}=\left\{\begin{array}{cc} s_{i} & \mathrm{if}\;iou\left(M,b_{i}\right)<N_{t}\\ 0 & \mathrm{if}\;iou\left(M,b_{i}\right)\geq N_{t} \end{array}\right. \end{array}$$

where $s_{i}$ signifies the confidence of the *i*th prediction box, *M* represents the box with the paramount confidence, $b_{i}$ denotes the *i*th prediction box, and $N_{t}$ is the predefined IoU threshold.

In densely populated scenarios, the conventional methodology risks neglecting pertinent prediction boxes. Soft-NMS ameliorates this by recalibrating, rather than discarding, boxes based on their IoU. The confidence of each prediction box is adjusted as:

(2) $$s_{i}=s_{i}\times f\left(iou\left(M,b_{i}\right)\right)$$

Here, f(·) is a function determining the change in confidence relative to IoU. Soft-NMS introduces two such functions: linear and Gaussian decay. The linear decay function is defined as f(x)=max(0,1−x), while Gaussian decay is expressed as $f\left(x\right)=\exp\left(-\sigma x^{2}\right)$.

Both linear and Gaussian decay strategies in Soft-NMS ensure that boxes with high IoU are adjusted but not discarded, improving adaptability in object detection.

**Figure 3:**
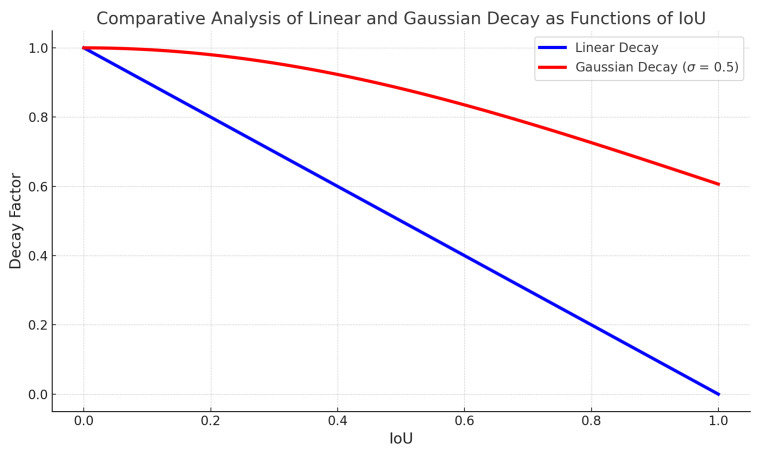
Comparison of linear and Gaussian decay in relation to IoU.

Figure 3 highlights the difference between the two decay methods. Linear decay shows a sharp reduction in confidence as IoU increases. In contrast, Gaussian decay provides a gentler decrease, suggesting boxes can be retained, even with high IoU. The choice between the two depends on application needs: linear for stricter overlap penalties and Gaussian for a milder approach. Soft-NMS offers more flexibility than traditional NMS, especially in dense object scenarios, by adjusting box confidence instead of outright removal.

Soft-NMS dynamically adjusts the score of detection boxes based on their IoU with the top-scoring box (*M*) as:

(3) $$s_{i}=s_{i}\times \exp\left(-\frac{iou{\left(M,b_{i}\right)}^{2}}{\sigma }\right)$$

where σ controls the decay rate. This method ensures that the scores of high-overlap boxes are reduced but the boxes are retained, enhancing object detection in packed scenarios.

In summary, Soft-NMS offers a refined approach to object detection, outperforming the rigid decisions of traditional NMS in dense contexts.

### 4.2. Involution Operator: A Paradigm Shift in Convolutional Neural Networks

In the domain of computer vision research, convolutional neural networks (CNNs) have been instrumental, largely credited to their convolution operator, which inherently offers spatial invariance and channel specificity [22]. While spatial invariance ensures parameter efficiency irrespective of spatial transformations, expanding the kernel size inflates the parameter count. This demands multilayer embeddings to extend the receptive field. However, stacking multiple small kernels does not match the efficiency of a larger kernel. Channel specificity, while preserving unique features within channels, can escalate model complexity due to distinct kernel weights.

The Involution operator [23] challenges conventional convolution by promoting spatial independence and channel sharing. Unlike convolution, the Involution operator focuses on individual pixels, minimizing redundancy and aiming to capture expansive contextual information.

Given an input feature map:

(4) $$F_{\mathrm{in}}=R^{H\times W\times C_{\mathrm{in}}}$$

and the corresponding output feature map:

(5) $$F_{\mathrm{out}}=R^{H\times W\times C_{\mathrm{out}}}$$

where $R^{H\times W\times C_{\mathrm{out}}}$ represents the entire pixel coordinate space, with *H* and *W* denoting the feature map’s height and width, respectively, and $C_{\mathrm{in}}$ and $C_{\mathrm{out}}$ signifying the number of input and output feature channels, respectively, the Involution operator segregates the channels into *G* distinct groups. Within each group, different spatial coordinates utilize unique kernels, maintaining orthogonality among channels in the same group. The custom Involution kernel for a pixel coordinate (i,j) is expressed as:

(6) $$I_{i,j}=\phi \left(F_{i,j}\right)=X_{1}\sigma \left(X_{0}F_{i,j}\right)$$

where ϕ() denotes the kernel generation function intrinsic to Involution, with $X_{0}$ and $X_{1}$ representing linear transformations. σ() is the activation function applied after batch normalization of the preceding linear transformations. Through these operations, the feature map for a specific pixel ($F_{i,j}^{\prime }$) is obtained. Using the reshape function, it aligns with the shape of the Involution kernel. This reshaped feature map undergoes multiplication and summation with the nearby feature vector on $F_{\mathrm{in}}$, culminating in $F_{\mathrm{out}}$.

**Figure 4:**
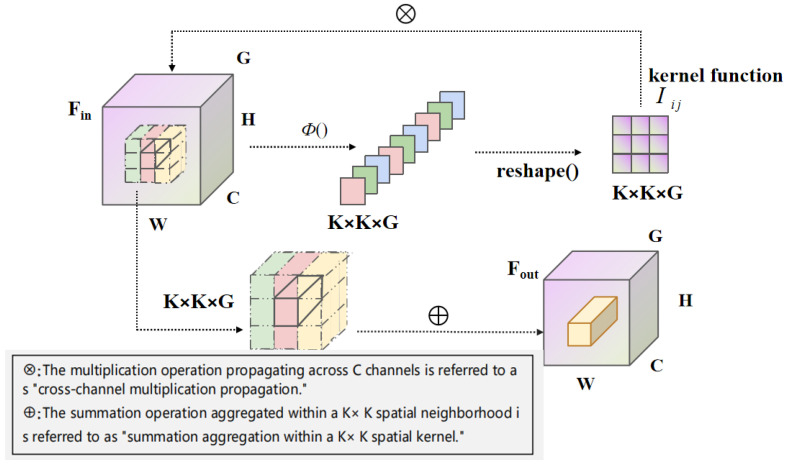
Principle and operation process of Involution.

The Involution mechanism, as presented in Figure 4, with its weight allocation across spatial positions, amalgamates contextual information across a broad spatial domain. This capability enables prioritization of salient visual cues within the image spatial domain, leading to reduced parameter overhead and faster convergence.

### 4.3. SE Attention Mechanism

The squeeze-and-excitation (SE) attention mechanism augments convolutional neural networks (CNNs) by modeling the interdependencies of feature channels. By doing so, the mechanism can weight input features, emphasizing channels of greater importance for specific tasks [24].

This procedure operates via two main stages: squeeze and excitation. In the squeeze stage, the spatial extents of the input feature map are compacted into a channel descriptor utilizing global average pooling, encompassing the overarching context. This descriptor then undergoes processing in the excitation stage via a duo of fully connected layers punctuated with a ReLU activation to infuse non-linearity.

**Figure 5:**
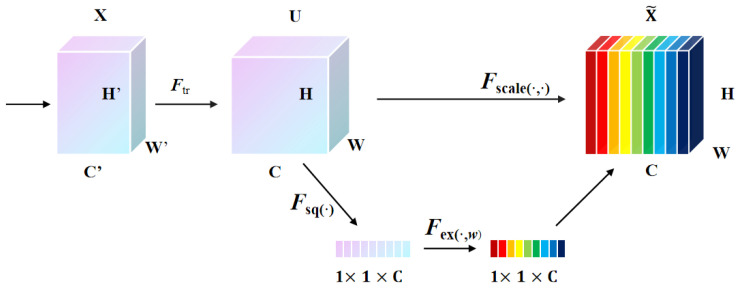
Structure and flow of the SE module.

Subsequent weights for every feature channel are consequently multiplied by the primary feature map, yielding a modulated feature map, as depicted in Figure 5. This illustration elucidates the configuration and progression of the SE module, with *W*, *H*, and *C* symbolizing the feature map’s width, height, and number of channels, respectively.

A primary merit of the SE approach is its seamless adaptability to current CNN designs. The negligible computational load makes it highly favorable for on-the-spot operations and mobile integrations. This method has consistently achieved remarkable results in a variety of computer vision endeavors.

### 4.4. Gsconv

With the continual advancement of convolutional neural networks (CNNs), there is an unceasing quest to bolster both efficiency and performance. This drive has catalyzed the development of lightweight models typified by architectures like MobileNet [25], ShuffleNet [26], and EfficientNet [27]. One standout technique facilitating such lightweight designs is depthwise separable convolution. This convolutional approach, which can be bifurcated into depthwise convolution (DWConv) and pointwise convolution (PWConv), offers a nuanced balance between computational efficacy and performance. Specifically, DWConv processes each channel of the input tensor independently using its own kernel, while PWConv, through its 1×1 kernels, undertakes dense computations, thereby merging information across channels.

Although DWConv, seen as a variant of grouped convolution, commendably reduces the number of parameters and computational demands, its insularity across channels can compromise computational accuracy. Conversely, while PWConv boosts accuracy by aggregating channel information, it introduces additional computational burdens. Conventional depthwise separable convolution, which juxtaposes DWConv and PWConv while keeping their outputs distinct, often faces a tradeoff: while it is more parameter-efficient than standard convolutions, it may not always deliver optimal performance.

**Figure 6:**
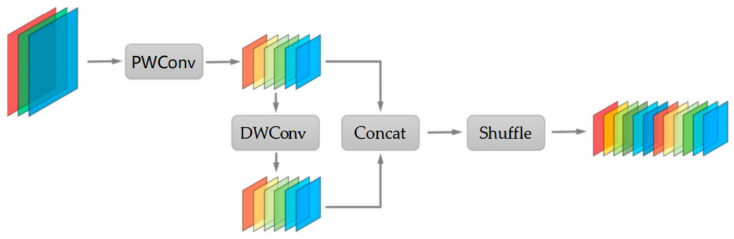
Structure and workflow of the GSConv module.

In an attempt to navigate these challenges, Li et al. unveiled the GSConv technique [28]. This innovative convolutional method illustrated in Figure 6 synergizes PWConv and DWConv in an intricate choreography. Initially, PWConv is employed on the input tensor, halving its channels. This intermediate tensor then undergoes DWConv. The culminating outputs from both these operations are concatenated, producing a tensor with enhanced channel richness. A subsequent shuffle operation across channels ensures a harmonious fusion of the two convolutional outputs.

Another convolutional paradigm worth noting is the Ghost-Shuffle convolution (GSConv). This method amalgamates the robustness of channel-dense convolution (SC) with the efficiency of channel-sparse convolution (DSC), striking a balance between computational speed and information retention.

Quantitatively, the computational demands of SC, DSC, and GSConv can be represented as:

(7) $${\mathrm{Time}}_{\mathrm{SC}}=W\times H\times K_{w}\times K_{h}\times C1\times C2$$

(8) $${\mathrm{Time}}_{\mathrm{DSC}}=W\times H\times K_{w}\times K_{h}\times 1\times C2$$

(9) $${\mathrm{Time}}_{\mathrm{GSConv}}=W\times H\times K_{w}\times K_{h}\times \frac{C2}{2}\times \left(C1+1\right)$$

where *W* and *H* symbolize the width and height of the feature map, respectively; $K_{w}$ and $K_{h}$ are the kernel dimensions; and C1 and C2 depict the input and output channel counts, respectively.

In summary, GSConv, through its intricate fusion of PWConv and DWConv, promises a future in which lightweight convolutional methods do not necessarily have to compromise on performance, heralding a new era in the domain of efficient deep learning architectures.

## 5. Experiment and Discussion

### 5.1. Dataset

Our study predominantly relies on the DOTA dataset [29]. DOTA stands out as a comprehensive dataset dedicated to object detection within aerial imagery. Its strength lies in its impressive volume and quality, especially when compared to counterparts of a similar nature. The dataset encapsulates aerial snapshots from varied sources: Google Earth, the JL-1 satellite, and the GF-2 satellite courtesy of the China Center for Resources Satellite Data and Application. These images capture objects varying in size, orientation, and configuration. Given the presence of low-resolution imagery and small-scale objects, object detection proves to be a demanding endeavor within this dataset. Each instance within DOTA is demarcated with a unique quadrilateral and falls under one of 15 prevalent object categories, such as airplanes, ships, storage tanks, and diverse sport facilities.

**Figure 7:**
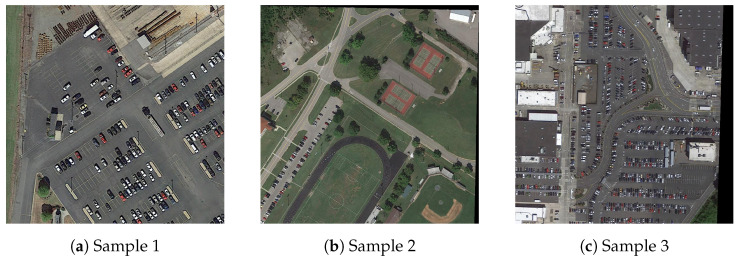
Some selected unprocessed images from the DOTA dataset.

For our research, we selected 6299 images from DOTA, which were systematically segmented into training, validation, and testing subsets in a 6:2:2 ratio. This collection, given its breadth and inherent challenges, serves as a rigorous test bed for our proposed methodology. A visual glimpse into the multifaceted nature of this dataset is provided in Figure 7.

### 5.2. Experiment and Parameter Setting

The setup for our experiments and specific parameter choices can be found in Table 1, Table 2 and Table 3.

**Table 1:** Environmental setup for experiments.

Experimental Component	Version/Model
OS	ubuntu20.04
Processor	Intel(R) Xeon(R) Gold 6330
Graphics card	RTX 3090
Python version	3.8
CUDA version	11.3
PyTorch version	1.10.0

- **OS**: The operating system used for the experiments, specifically Ubuntu version 20.04;
- **Processor**: The CPU type, namely an Intel Xeon Gold 6330;
- **Graphics card**: The GPU model utilized for the experiments, i.e., RTX 3090;
- **Python version**: The version of the Python programming language deployed;
- **CUDA version**: The version of NVIDIA’s parallel computing platform and application programming interface;
- **PyTorch version**: The version of the open-source machine learning library PyTorch used.

**Table 2:** Parameters for model training.

Parameter Name	Chosen Setting
Image dimensions	640 × 640
Batch Size	36
Number of epochs	500
Data augmentation method	Mosaic

- **Image dimensions**: The size of input images resized to 640 × 640 pixels;
- **Batch size**: The number of training examples utilized in one iteration;
- **Number of epochs**: The total number of forward and backward passes of all training examples.
- **Data augmentation method**: The technique deployed to augment the training dataset.

**Table 3:** Hyperparameters for training.

Hyperparameter	Value
Initial learning rate (Lr0)	0.01
Final learning rate (Lrf)	0.01
Momentum	0.937
Weight decay coefficient	0.0005

- **Initial learning rate (Lr0)**: The starting learning rate, determining the step size at each iteration;
- **Final learning rate (Lrf)**: The learning rate used during the final epoch.
- **Momentum**: A hyperparameter aiding in accelerating gradient vectors, optimizing convergence;
- **Weight decay coefficient**: A regularization term added to avert overfitting.

### 5.3. Metrics for Evaluating Detection Accuracy

The performance of the detection technique was assessed based on three fundamental metrics: detection precision, parameter count, and computational complexity. Detection precision measures the skill of the algorithm in identifying and classifying entities. In this context, we used the mean average precision (mAP) with an intersection over union (IoU) benchmark set at 0.5 for our assessment standard. Mathematically, mAP is expressed as:

(10) $$mAP=\frac{1}{n}\sum_{i=1}^{n}AP(i)$$

In this context, *n* denotes the entire category count. Given that our dataset comprises 15 distinct classes, *n* is set to 15. For any specified category, the average precision (AP) is calculated as the mean precision across varied recall thresholds:

(11) $$P=\frac{TP}{TP+FP}$$

(12) $$R=\frac{TP}{FN+TP}$$

(13) $$AP=\int_{1}^{0}P(R)dR$$

In the above equations, *P* denotes precision, which represents the likelihood of accurate prediction among all samples predicted as positive; *R* stands for recall, representing the likelihood of detecting all positive samples; and true positives, true negatives, false positives, and false negatives are denoted by TP, TN, FP, and FN, respectively.

Computational complexity is assessed using GFLOPs, shedding light on the model’s operational demands and efficiency. This measure is pivotal for scenarios demanding instantaneous processing or for use on devices constrained in terms of computational power. The term GFLOPs denotes the quantity of floating-point computations executed every second, acting as a primary indicator for appraisal of the model’s computational prowess. Moreover, the count of Parameters offers a vital gauge to ascertain the model’s size and efficacy and is directly linked with the computational overhead and memory prerequisites of the model.

### 5.4. Examination of Experimental Results

The research outlined in this paper is structured around three primary experimental clusters:

1. **Improvement verification experiment**: In this experiment, the proposed improvements are tested rigorously to ensure that they bring about the desired enhancement in performance. This serves as a validation step, substantiating the relevance and utility of the proposed changes.
2. **Relative performance evaluation of enhanced algorithms**: This study aims to evaluate the performance of algorithms after they have been enhanced, providing a clear benchmark for their effectiveness and improvements over the baseline or previous versions.
3. **Comparative tests on attention methodologies**: This cluster of experiments delves into various attention mechanisms, comparing their performances and extracting insights with respect to which mechanisms are most well suited for the task at hand. By doing so, we aim to uncover the nuances and intricacies of different attention models in the context of our research problem.

#### 5.4.1. Improvement Verification Experiment

In this study, we explore four distinct modifications based on the YOLOv5 architecture: the integration of Involution, GSConv, SE, and Soft-NMS. By contrasting the performance of these modifications against the original YOLOv5, we aim to identify the optimal model structure for specific tasks.

Based on the results reported in Table 4, we can draw the following conclusions.

1. **Model parameters**: Both *YOLOv5 + Involution* and *YOLOv5 + GSConv* demonstrate a reduction in the number of parameters compared to the original YOLOv5. This suggests that these models might be more lightweight and suitable for deployment in resource-constrained environments.
2. **Computational complexity**: The GFLOPs for all models lie in the range of 15.0 to 15.9, indicating that the modifications did not significantly alter the computational demands of the model.
3. **Performance metrics**:
  - In terms of precision (P), *YOLOv5 + SE* outperforms the other models, reaching 87.4%. This suggests that the SE attention mechanism can enhance the model’s predictive accuracy.
  - Both *YOLOv5 + Involution* and *YOLOv5 + GSConv* show improvements in recall (R), implying that these strategies might aid the model in detecting a higher number of objects.
  - In terms of mAP50, *YOLOv5 + Soft-NMS* stands out, with a performance of 77.6%, which is significantly higher than that of the other variants. This indicates that Soft-NMS might be more effective in eliminating redundant detection boxes.

In summary, each modification offers advantages in certain aspects over the original YOLOv5.

#### 5.4.2. Relative Performance Evaluation of Enhanced Algorithms

We integrated the specified enhancements into the YOLOv5 framework and juxtaposed it against the unaltered YOLOv5 setup. The outcomes of these experiments are delineated in Table 5.

According to the presented results, SenseLite outperforms the standard YOLOv5. Specifically, SenseLite reduces the number of parameters by 30.5% and the computational complexity from 15.9 GFLOPs to 11.2. Moreover, it achieves a precision of 85.4%, a recall rate of 70.8%, and an mAP50 of 78.3%.

**Figure 8:**
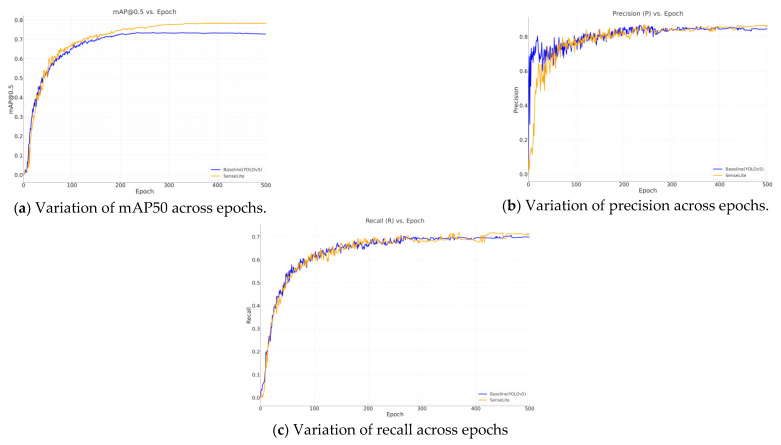
Metric analysis across epochs using the DOTA dataset.

The progress of these metrics over epochs is visually illustrated in Figure 8. The charts indicate that as training progresses:

- The mAP50 (Figure 8a) exhibits a steady increase, reflecting the model’s enhanced ability to detect objects with a higher degree of overlap with ground-truth boxes;
- Precision (Figure 8b) follows an upward trajectory, signifying the model’s increasing accuracy in object localization;
- Recall (Figure 8c), despite minor fluctuations, generally trends upward, indicating the model’s growing capability to identify a larger fraction of present objects.

These trends further underscore the efficacy of the SenseLite model, establishing it as a more efficient and accurate alternative for object detection tasks.

#### 5.4.3. Comparison Experiments for Different Attention Mechanisms

To comprehensively evaluate the squeeze-and-excitation (SE) attention mechanism, we performed a side-by-side comparison with other eminent attention structures, including:

- **Convolutional block attention module (CBAM)** [30]: CBAM focuses on boosting the representational power of convolutional features by adapting both spatial and channel-wise attention.
- **Coordinate attention (CA)** [31]: CA supports the model in capturing long-range dependencies by leveraging the coordinate information of features.
- **Efficient channel attention (ECA)** [32]: ECA captures channel-wise dependencies more efficiently by focusing on local neighborhoods in the channel dimension.

From Table 6, it is evident that the squeeze-and-excitation (SE) mechanism employed in this study has a similar number of parameters as other mechanisms. However, it outperforms them in terms of precision (P) and overall object detection accuracy. Therefore, in this study, we chose to utilize the SE attention mechanism.

### 5.5. Ablation Experiment

To rigorously evaluate the contributions of the enhancements in proposed in this study for small object detection within aerial images, we meticulously conducted a series of ablation studies. The delineation is as follows: (1) Baseline (YOLOv5): The foundational model, YOLOv5, with no additional modules; (2) YOLO + SE: YOLOv5 incorporated with a squeeze-and-excitation (SE) module designed to exploit channel interdependencies; (3) YOLO + Soft-NMS: YOLOv5 equipped with Soft-NMS, an advanced mechanism to manage overlapping bounding boxes; (4) YOLO + GSConv: The YOLOv5 model is augmented with GSConv, a lightweight convolution technique aimed at streamlining the model without compromising performance; (5) YOLO + Involution: YOLOv5 integrated with the Involution operation, which facilitates the capture of richer contextual information across a more extensive spatial domain; (6) YOLO + Soft-NMS + GSConv: An amalgamation of YOLOv5 with both Soft-NMS and GSConv; (7) YOLO + Soft-NMS + GSConv + Involution: An enriched version of YOLOv5 integrating Soft-NMS, GSConv, and the Involution operation; (8) SenseLite (full model): Our holistic model that synergistically combines all the aforementioned modules.

**Figure 9:**
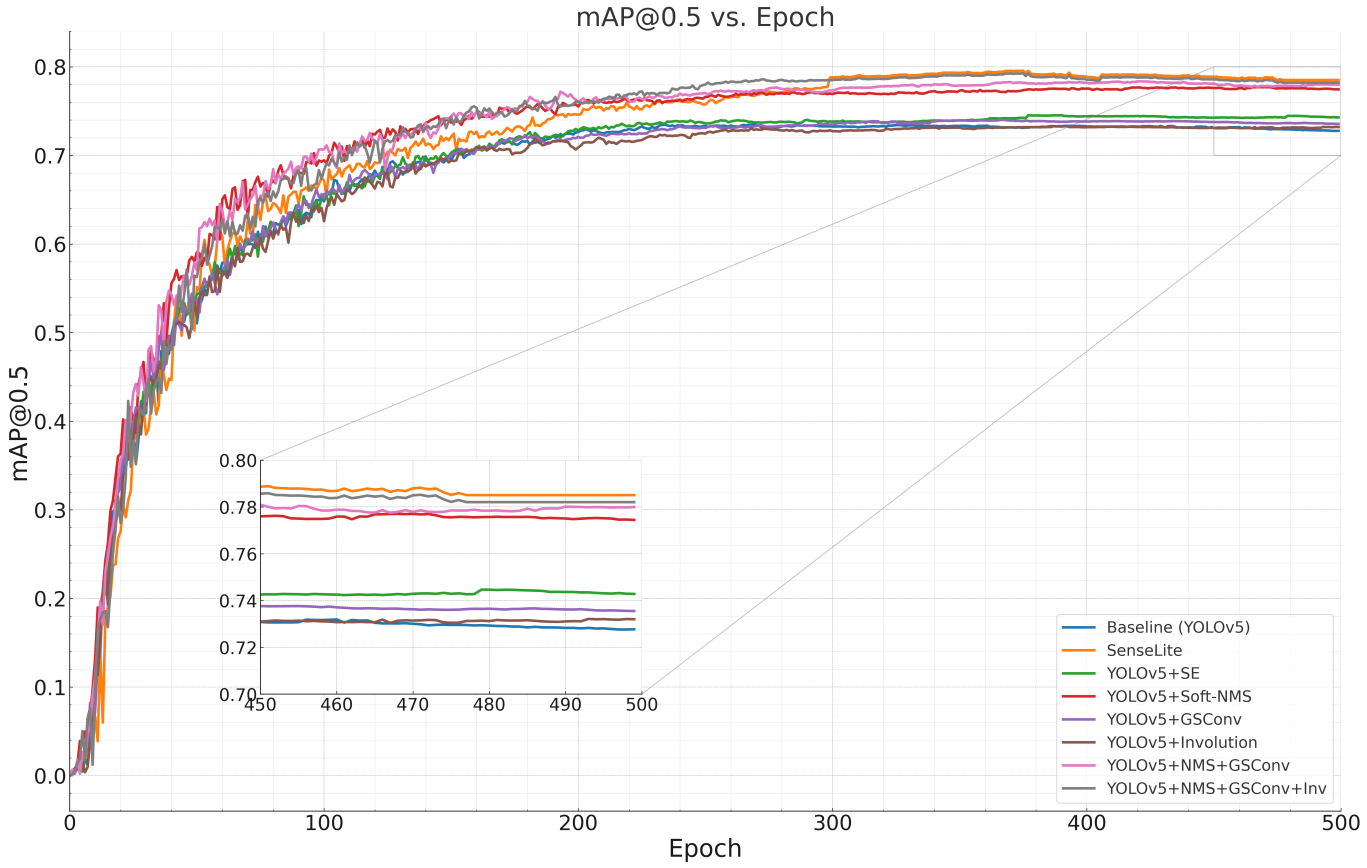
mAP values of different modules.

Every test was conducted on the DOTA dataset, maintaining a uniform input resolution of 640 × 640. Table 7 showcases the performance metrics from the ablation studies. Figure 9 showcases the mAP@0.5 from the ablation studies.

Table 7 offers a detailed breakdown of the outcomes from our experiments.

The baseline (YOLOv5) represents our foundational model, achieving an mAP50 of 72.8%. With a parameter count of 7.05 million and a computational load of 15.9 GFLOPs, it sets the stage for our subsequent modifications. Introducing Soft-NMS (SN) to the YOLO architecture leads to a 4.8% surge in mAP50, validating its efficacy in curbing false positives in regions with overlapping bounding boxes. This adjustment is particularly crucial for aerial images, where objects are closely packed and often overlap. The integration of the squeeze-and-excitation (SE) module takes the precision up a notch to 87.4%, a clear testament to its capability of fine tuning channel-wise feature recalibrations. This enhancement ensures our model responds robustly to the intricate features of small objects. The addition of GSConv stands out with a dual impact. While it trims down the model parameters to 6.61 million and computational complexity to 15.3 GFLOPs, the mAP50 witnesses a modest increase to 73.6%. This suggests that GSConv’s lightweight convolution mechanism not only makes the model leaner but also slightly more efficient in detection tasks. Involution, when added to our YOLO model, captures more contextual information across an expansive space, causing a minor dip in precision to 85.5%. However, this is counterbalanced by its broader contextual understanding, as reflected by an mAP50 of 73.1%. Merging Soft-NMS and GSConv creates a blend that pushes the mAP50 up to 78.3%. The model remains lightweight, with the number of parameters still hovering around 6.61 million, reinforcing the idea that strategic combinations can achieve better detection without burdening the computational resources. When Involution joins the fray with Soft-NMS and GSConv, it crafts a more nuanced model. The number of parameters drops dramatically to 4.89 million, and GFLOPs come down to 11.2, yet the mAP50 inches up to 78.2%. This configuration reveals that the inclusion of Involution, while beneficial for model lightness, demands a careful balancing act to harness its full potential for detection. Finally, SenseLite, our pièce de résistance, incorporates all modules. The model’s efficiency shines with a mere 4.90 million parameters and 11.2 GFLOPs. The mAP50 peaks at 78.3%, underscoring that the harmonized synergy of all modules is indispensable for achieving top-tier detection performance in aerial images.

In essence, our ablation study affirms that while each module has its unique strengths, their orchestrated interplay is what propels SenseLite to its zenith of performance.

### 5.6. Evaluation on Specialized Small Object Detection Datasets

In order to provide a comprehensive assessment of the SenseLite method on small object detection tasks, we incorporated two specialized datasets: AI-TOD and SODA-A.

#### 5.6.1. Evaluation on AI-TOD Dataset

AI-TOD (Artificial Intelligence Tiny Object Detection) is a dataset specifically crafted for the detection of tiny objects [33]. It encompasses a variety of scenes and backgrounds, such as urban landscapes, rural settings, and maritime environments, offering a rich data source for small object detection research. It includes:

- Over 10,000 high-resolution images;
- Diverse scenes including urban landscapes, rural settings, and maritime environments;
- Eight distinct object categories: ‘airplane’, ‘bridge’, ‘storage-tank’, ‘ship’, ‘swimming-pool’, ‘vehicle’, ‘person’, and ’wind-mill’;
- Annotations with bounding boxes and class labels;
- An average object size of less than 32 × 32 pixels, emphasizing the challenge of detecting tiny objects.

#### 5.6.2. Evaluation on SODA-A Dataset

SODA-A [34], which is specifically tailored for detecting small objects in aerial images, offers an array of challenges, from densely populated object clusters to varying object scales.

- Over 2000 aerial images captured under diverse lighting and weather conditions;
- Challenges such as densely populated object clusters, varying object scales, and occlusions;
- Ten distinct object categories: ‘airplane’, ‘helicopter’, ‘small-vehicle’, ‘large-vehicle’, ‘ship’, ‘container’, ‘storage-tank’, ‘swimming-pool’, ‘windmill’, and ‘ignore’;
- Comprehensive annotations, including bounding boxes, class labels, and occlusion flags.

From the Table 8 and Table 9, it is evident that the proposed SenseLite model consistently surpasses the baseline YOLOv5s model on both datasets. The structural enhancements in SenseLite contribute to its superior performance, reflecting its adaptability and potency in detecting small objects across diverse aerial datasets.

### 5.7. Benchmarking against Leading Algorithms

To ascertain the efficacy of the enhanced algorithm presented in this work, we undertook a comprehensive experimental assessment. We juxtaposed our method against prominent detection algorithms, namely SSD, Fast R-CNN, Faster R-CNN, RetinaNet, YOLOv3, YOLOv4, and YOLOv5. For context, our implementation of Faster R-CNN employs ResNet-101 as its backbone, whereas SSD leverages ResNet-50. Both algorithms were fed inputs, maintaining uniform dimensions in terms of width and height. In Table 10, we present the results of our comparisons.

From the data delineated in Table 10, it becomes clear that the introduced SenseLite technique holds an edge over other leading algorithms concerning precision (P%), recall (R%), and mean average precision (mAP%). Notably, SenseLite registers an mAP of 78.3%, marking a noteworthy advancement from YOLOv5’s 72.8%. This 5.5% surge in mAP accentuates the prowess and resilience of the SenseLite framework, particularly in managing intricate object identification challenges.

When comparing the precision metrics, SenseLite (85.4%) surpasses the second-best, YOLOv5, by nearly 1%. This improvement highlights the model’s ability to correctly identify positive instances, reducing the chances of false positives. In terms of recall, SenseLite again leads the pack, with 70.8%, emphasizing its capability of detecting most of the actual positive samples, thereby minimizing missed detections.

While YOLOv4 and YOLOv5 exhibit commendable performances, the integration of advanced modules and optimization techniques in SenseLite enables it to set a new benchmark in the domain. Its consistently superior performance across all metrics solidifies the effectiveness of the SenseLite algorithm in achieving a balance between precision and recall while ensuring high detection accuracy.

### 5.8. Visualization of Experimental Results

In order to emphasize the effectiveness of our proposed model, SenseLite, we compared its performance with that of the standard YOLOv5. The ensuing visualizations clearly depict the technological advancements achieved through our modifications. The SenseLite model distinctly showcases superior detection capabilities, especially when detecting small and intricate objects. From side-by-side comparisons, it is evident that, in certain instances, SenseLite can more accurately identify objects that the standard YOLOv5 might overlook or misclassify. This superiority is largely attributed to the advanced features and optimizations integrated into SenseLite.

**Figure 10:**
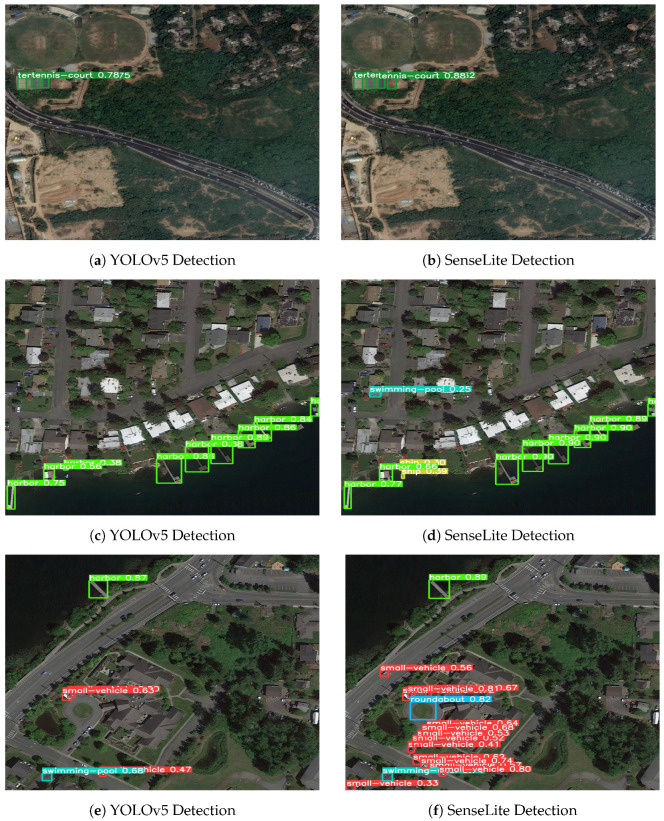
Comparative visualization between standard YOLOv5 detections and our improved SenseLite detections. As can be observed, SenseLite consistently offers enhanced detection accuracy across different scenarios.

Delving deeper into the comparison results presented in Figure 10, we observe that SenseLite consistently detects minute or detail-rich objects across multiple test scenarios—objects that might be overlooked when using YOLOv5. In comparison to YOLOv5, SenseLite exhibits better performance in terms of false positives and false negatives, signifying not only its ability to detect objects accurately but also its reduced rate of erroneous detections. Moreover, SenseLite maintains stable and efficient performance across a variety of different scenes. Even with its enhanced detection capabilities, its real-time performance remains largely unaffected, making it an ideal choice for applications that demand quick responses. This comprehensive analysis affirms that our SenseLite surpasses the standard YOLOv5 in multiple key areas—accuracy, adaptability, and real-time performance—demonstrating exceptional prowess and validating the success of our improvements.

## 6. Conclusions

Detecting small objects in aerial imagery presents unique challenges due to the inherently low resolution and intricate nature of the backgrounds. Addressing these challenges, we introduced SenseLite, an optimized model based on YOLOv5 tailored specifically for small object detection within aerial imagery.

A conglomerate of techniques was employed to elevate detection accuracy and efficiency. The integration of Involution offered a novel mechanism to gather extensive contextual semantic information while ensuring adaptive weight distribution across varying image locales. By harnessing GSConv, our model underwent a simplification, slashing its complexity without compromising detection performance, making it apt for swift predictions. Furthermore, the incorporation of a squeeze-and-excitation (SE) attention module enabled the effective utilization of information spanning diverse channels, while Soft-NMS addressed overlapping bounding box concerns.

One notable achievement of SenseLite is its significant reduction in network parameters down to 4.9 M, corresponding to a decrease of approximately 30.5 %. This streamlined model structure, coupled with reduced computational complexity (GFLOPs slashed from 15.9 to 11.2), positions it as a formidable contender in aerial image detection tasks. Despite its lean architecture, SenseLite surpasses the foundational YOLOv5 framework when it comes to efficacy. Rigorous experimentation using the DOTA dataset further corroborated the prowess of SenseLite, registering a 5.5% elevation in mAP in comparison to YOLOv5.

However, we acknowledge certain limitations of SenseLite. Its performance might be compromised in scenarios with substantial obstructions or intricate backgrounds, and it may not meet real-time detection needs on some devices. Challenges in detecting objects of diverse scales and poses, domain adaptability in unfamiliar settings, and potential data imbalance issues in certain categories are areas we aim to address in future research.

Peering into the future, we aspire to further research and enhance SenseLite. Prospective directions encompass the assimilation of advanced attention mechanisms, innovative training strategies, and refinement of the model architecture. Our ultimate goal remains steadfast: to set SenseLite as the benchmark for small object detection in aerial imagery, continually enhancing its performance across varied scenarios.

## Figures and Tables

**Table 4:** Validation of different attention mechanisms.

Algorithm	Parameter (M)	GFLOPs	P (%)	R (%)	mAP50 (%)
YOLOv5	7.05	15.9	84.5	69.4	72.8
YOLOv5 + Involution	6.33	15.0	86.1	70.1	73.6
YOLOv5 + GSConv	6.61	15.3	86.1	70.1	73.6
YOLOv5 + SE	7.07	15.9	87.4	69.1	74.5
YOlOv5 + Soft-NMS	7.05	15.9	86.5	69.1	77.6

**Table 5:** Comparison of algorithms based on performance metrics.

Model	Parameters (M)	GFLOPs	Precision (%)	Recall (%)	mAP50 (%)
YOLOv5s	7.05	15.9	84.5	69.4	72.8
SenseLite	4.90	11.2	85.4	70.8	78.3

**Table 6:** Validation of Different attention mechanisms.

Algorithm	Parameters (M)	GFLOPs	P (%)	R (%)	mAP50 (%)
YOLOv5 + CA	7.07	15.9	86.7	68.5	73.5
YOLOv5 + ECA	7.05	15.9	81.5	71.2	74.4
YOLOv5 + CBAM	7.07	15.9	86.6	68.5	73.0
YOLOv5 + SE	7.07	15.9	87.4	69.1	74.5

**Table 7:** Comparison of the results of ablation studies.

Model	SE	Soft-NMS	GSConv	Involution	Parameters (M)	GFLOPs	P (%)	R (%)	mAP50 (%)
Baseline (YOLOv5)					7.05	15.9	84.5	69.4	72.8
YOLO + SE	✓				7.07	15.9	87.4	69.1	74.5
YOLO + SN		✓			7.05	15.9	86.5	69.1	77.6
YOLO + GS			✓		6.61	15.3	86.1	70.1	73.6
YOLO + Inv				✓	6.33	15.0	85.5	69.2	73.1
YOLO + SN + GS		✓	✓		6.61	15.3	82.1	70.5	78.3
YOLO + SN + GS + Inv		✓	✓	✓	4.89	11.2	83.6	70.4	78.2
SenseLite	✓	✓	✓	✓	4.90	11.2	85.4	70.8	78.3

**Table 8:** Comparison of YOLOv5s and SenseLite on the AI-TOD dataset.

Model	Parameters (M)	GFLOPs	Precision (%)	Recall (%)	mAP50 (%)
YOLOv5s	7.03	15.8	61.7	25.7	24.1
SenseLite	4.88	11.1	52.2	22.9	29.1

**Table 9:** Comparison of YOLOv5s and SenseLite on the SODA-A dataset.

Model	Parameters (M)	GFLOPs	Precision (%)	Recall (%)	mAP50 (%)
YOLOv5s	7.03	15.8	27.2	13.5	20.3
SenseLite	4.90	11.3	30.2	15.3	24.1

**Table 10:** Comparison with advanced methods.

Method	Precision%	Recall%	mAP%
SSD	68.4	56.2	59.6
RetinalNet	71.4	59.5	62.2
Fast R-CNN	73.8	54.1	60.4
Faster R-CNN	76.7	58.3	62.6
YOLOv3	77.8	61.3	62.2
YOLOv4	81.3	67.6	69.4
YOLOv5	84.5	69.4	72.8
SenseLite	85.4	70.8	78.3

## Data Availability

Not applicable.
